# Morphogenetic peculiarities of reproductive biology in sugar beet (Beta vulgaris L.) breeding

**DOI:** 10.18699/VJGB-23-27

**Published:** 2023-06

**Authors:** T.P. Zhuzhzhalova, A.A. Nalbandyan, E.N. Vasilchenko, N.N. Cherkasova

**Affiliations:** The A.L. Mazlumov All-Russian Research Institute of Sugar Beet and Sugar, vil. VNIISS, Ramonsky district, Voronezh region, Russia; The A.L. Mazlumov All-Russian Research Institute of Sugar Beet and Sugar, vil. VNIISS, Ramonsky district, Voronezh region, Russia; The A.L. Mazlumov All-Russian Research Institute of Sugar Beet and Sugar, vil. VNIISS, Ramonsky district, Voronezh region, Russia; The A.L. Mazlumov All-Russian Research Institute of Sugar Beet and Sugar, vil. VNIISS, Ramonsky district, Voronezh region, Russia

**Keywords:** sugar beet, reproduction, morphogenesis, embryoidogeny, molecular markers, self-incompatibility, cytoplasmic male sterility (CMS), apomixis, сахарная свекла, репродукция, морфогенез, эмбриоидогения, молекулярные маркеры, самонесовместимость, цитоплазматическая мужская стерильность (ЦМС), апомиксис

## Abstract

This review considers the processes of morphogenesis used in the development of propagation methods and the creation of a new starting material for sugar beet. It has been demonstrated that methods of particulation, in vitro microcloning and cell breeding that reflect non-sexual forms of plant reproduction increase the effectiveness of breeding experiments. The review describes the in vitro culture methods maintaing a tendency in plants for vegetative propagation and stimulating increase in genetic variability of properties when mutagens such as ethyl methanesulfonate, alien genetic structures with mf2 and mf3 bacterial genes in Agrobacterium tumefaciens strains, and selective agents (Сd++ ions and abscisic acid) are incorporated into plant cells. It presents the results of using fluorescent microscopy, cytophotometry, biochemical analysis and determining the level of phytohormones and content of nucleic acids in nuclei for forecasting the seed setting ability. It has demonstrated that long self-pollination of plants causes decrease in fertility of pollen grains, resulting in the sterilization of male gametes and the appearance of pistillody flowers. Self-fertile plants isolated from these lines serve as sterility fixers, while the apomixis elements increased the ovule number, additional embryo sacs and embryos. A role of apomixis in contributing to variability in the onto- and phylogenetic development of plants have been substantiated. The review reflects the morphological features of the in vitro development of sexual and somatic cells in embryos during the formation of seedlings based on floral and vegetative embryoidogeny. Use of the SNP and SSR (Unigenes) molecular-genetic markers having a high polymorphism level has appeared effective to characterize the developed breeding material and hybrid components when carrying out crossings. The study of sugar beet starting materials for the presence of TRs mini-satellite loci making it possible to reveal O-type plants-pollinators (sterility fixing agent) and MS-form plants are of interest for breeding as well. The selected material can be widely used in breeding to produce hybrids, allowing for a 2–3- fold reduction of the development period. The review also discusses the prospects for the development and implementation of new methods and original schemes in sugar beet genetics, biotechnology and breeding.

## Abstract (ru)

В обзоре рассмотрены процессы морфогенеза, задействованные при разработке методов размножения и создания нового исходного материала сахарной свеклы. Показано, что методы партикуляции, микроклонирования in vitro и клеточной селекции, отражая неполовые формы репродукции растений, повышают результативность селекционных экспериментов. Приведены данные применения методов культуры in vitro, сохраняющих склонность растений к вегетативному размножению и стимулирующих повышение генетической изменчивости при использовании мутагенов этилметансульфоната, методов генетической трансформации (бактериальные гены mf2 и mf3) и селективных агентов (ионы Сd++ и абсцизовая кислота). Отражены результаты применения методов оценки степени самонесовместимости растений: флуоресцентной микроскопии, цитофотометрии, и определения уровня фитогормонов и содержания нуклеиновых кислот в ядрах клеток для прогнозирования завязываемости семян. Выявлено, что длительное самоопыление растений вызывает снижение фертильности пыльцевых зерен, стерилизацию мужских гамет и образование пистилодийных цветков. Самофертильные растения, выделенные из этих линий, служат закрепителями сте- рильности. В обзоре продемонстрирована роль апомиксиса в реализации изменчивости при онто- и филоге- нетическом развитии растений. Отражены морфологические особенности развития половых и соматических клеток зародышей in vitro при формировании проростков на основе флоральной и вегетативной эмбриоидогении. Применение молекулярно-генетических маркеров SNP и SSR, обладающих высоким уровнем по- лиморфизма, оказалось эффективным для характеристики создаваемого селекционного материала гибридов при проведении скрещиваний. Интерес для селекции представляют исследования исходных материалов са- харной свеклы на наличие минисателлитных локусов TRs, позволившие выявлять растения-опылители О-типа (закрепитель стерильности) и растения МС (мужскостерильные) формы. Созданный материал может найти широкое использование в селекционной работе при создании гибридных форм, сокращая сроки селекции в 2–3 раза. Обсуждаются перспективы разработки и возможности применения новых методов и оригинальных схем в генетике, биотехнологии и селекции сахарной свеклы.

## Introduction

The first studies into the reproductive properties of sugar beet date back to the early ХХ century, when the systematic characterization of the species was carried out and its evolutionary paths described. It was also when the investigation of embryological patterns and morphological properties of plant cells, organs, and tissues started. The researchers began to focus on the developmental nuances of sugar beet flowers, anthers, and pollen grains. Studies into anatomical and embryological development patterns of ovules, embryos, and seeds were initiated, handbooks of anatomical charts for Beta vulgaris representing the key morphological traits of the species were prepared. Later, more in-depth studies focused on formation of flowers and their elements, reduction division stages, pollen development stages, embryo sac development, as well as embryo and seed formation. These efforts made possible the large-scale research work on genetics and breeding of sugar beet, laid foundation for genetic breeding programs, and eventually stimulated the creation of the first multigerm sugar beet cultivars (Mazlumov, 1970).

The late ХХ century marked a transition from population breeding to hybrid breeding for cytoplasmic male sterility (CMS), monogermity, and increased ploidy level. This major shift was facilitated by the results of studies into karyotype structure of the genus Beta, mitotic peculiarities, development of generative organs, as well as fertilization and embryogenesis in development of polyploids and self-pollinated lines. These findings showed feasibility of heterosis breeding for cultivation of hybrids with high crop yields and sugar contents (Balkov et al., 2017). Cultivation of high-yielding genotypes with valuable traits was also driven by the studies of reproductive processes ensuring genetic variability based on morphological, cytological, and biochemical traits (Simko et al., 2012; Stеvanato et al., 2015).

Reproductive biology studies the development of plant organisms at molecular and subcellular, cellular, organismic, and population levels to determine how their traits are propagated to the next generation (Maletskiy, 2005). This is why sugar beet breeding material is analyzed using a wide range of methods intended for various reproduction types, including genetic markers (Banzal et al., 2014), cytology, embryology, physiology, and morphology (Paesold et al., 2012; Tomaszevska- Sowa et al., 2017). The studies are designed taking into account the nuances of plant reproduction having to do with multiple variants of procreation (sexual, asexual, and apomictic), various morphogenetic pathways (embryogenesis, embryoidogenesis, and hemmorhizogenesis), and reproduction types (seed or vegetative) (Batygina, Vinogradova, 2007). This approach makes it possible to predict the progeny genotype and shows new opportunities for controlling specific ontogenetic stages and designing new breeding programs (Maletskaya, Maletskiy, 2010; Bartenev et al., 2018).

Genetic marker methods appear to be the most common techniques in plant breeding today. The high occurrence rate of molecular markers in the genome, studied using the universal and continuously evolving analytical methods makes the use of DNA markers viable for breeding purposes (Khlestkina, 2013). They make it possible to not only accurately and quickly identify genetic variability in sugar beet populations, lines, and hybrids, but also determine the breeding value of the experimentally obtained plant material (Nalbandyan et al., 2020). Molecular markers ISSR (Inter-Simple Sequence Repeats), SSR (Simple Sequence Repeats), and SNP (Single Nucleotide Polymorphism) are considered the most efficient.

The goal of the present paper is to review the data and methods (both proprietary and available in the literature) used for characterization of the morphogenetic potential of sugar beet at various development stages.

## Reproduction of vegetative plant tissues

Particulation. A common way to fix the most valuable traits and attributes of sugar beet breeding material is to use the plant’s capability of vegetative reproduction, which is due to the morphological and anatomical peculiarities of the roots, stems, and shoot buds, as well as to their ontogenetic transformation.

Growth and development processes in plants after seed germination include root and leaf system formation. At this stage, the crown, i. e. the upper part of the root, represents a reduced dome-shaped stem with a rosette leaf arrangement. The apical bud is located at the center of the rosette, and the axillary buds formed by organogenesis at the base of leaves.

A successful vegetative reproduction in winter is ensured by preliminary vernalization promoting epigenetic changes in the gene expression driving the post-vernalization development of plants (Shevelukha, 1992; Lutova et al., 2010). The observed activation of meristematic tissues (phellogen and cambium) stimulating the development of apical and axillary buds and then central and lateral shoots ensures rapid reproduction of the breeding material through traumatic particulation (Barykina et al., 2004). Here, the selected super-elite roots (pedigree) were divided into 4–8 groups, with one group cloned, and the rest grown for seeds

The main shortcomings of the method were the significant time it took to achieve material uniformity (up to 5 years), the limited number of reproduced clones, and high labor intensity. At the same time, this approach made it possible to preserve the genotypes with high crop yields and sugar contents relatively pure for several generations and thus found its use in breeding schemes based on individual selection combined with cloning and hybridization (Balkov et al., 2017).

The reproduction process was later intensified by cloning the plants using rooted lateral shoots (with 3–8 leaves) developing from axillary buds. The method ensured a significant increase in number of rooted shoots and grown plants, but gained no traction in sugar beet breeding.

The particulation ability of sugar beet is a backup reproduction mechanism, which combined with seed reproduction ensures the organism’s flexibility, survival, and overall preservation of the B. vulgaris species.

Microcloning. Biotechnology methods for mass vegetative reproduction in vitro appear to show the most promise in breeding (Murashige, 1974). These methods rest upon the ability of somatic cells to recover the organism’s continuity with all its traits while cultivation (Atanasov, 1993).

It was found that a plant can develop from vegetative cells in two ways: either formed from callus tissue through regeneration of buds and embryoids or from apical meristems, axillary and dormant buds as a result of activation processes (Katayeva, Butenko, 1983; Rusea et al., 2020). However, long-term subculturing of the callus led to changes in ploidy, accumulation of genetic mutations, and loss of morphological potential. Shoot meristems, laminae, petioles, anthers, ovules, embryos, and seeds are the most common explants for micropropagation (Leyke et al., 1980). Hormonal activation of this material leads to the development of shoot buds and seedlings (Ilyenko, 1983; Seman, Farago, 1988).

The most convenient way to refine sugar beet microcloning process is through the use of apical meristem due to its cells’ high genetic stability and high morphogenetic ability (Znamenskaya, 2010). Apical meristem is responsible for creation of the above-ground organs of plants, developing in a modular fashion (a shoot element includes a leaf, an internode, and an axillary bud), and meristematic stem cells can differentiate and stimulate the development of new organs (Lutova et al., 2010). Another linchpin of the method is totipotency, a unique property of plant cells allowing them to recreate a plant organism with all of its traits, when isolated from a parent organism and affected by exogenous factors of a culture medium (Shevelukha, 1992).

Cultivation of the starting material for explantation under cover, which made the contamination of explants 1.5 times as low, was a necessary condition for microcloning refinement (Znamenskaya, Zhuzhzhalova, 1989). The next mandatory stage of the cultivation process was using the optimized sterilization protocol for apical explant meristems that were soaked in 0.05 % corrosive sublimate solution for 1–3 hours.

Maintaining the optimal hormonal balance of 0.2– 0.3 mg/l of gibberellin, 6-benzylaminopurine (BAP), and kinetin (1:1:1) in the culture medium stimulated morphogenesis. Root formation was induced by adding 1 mg/l of indole butyric and indole acetic acids into the medium. With these contents, the number of lateral shoots per explant increased to 9–10, microclone height – to 73.4 mm, and root formation – to 74.3 %. This method made it possible to obtain an unlimited number of clones from one donor by halving the breeding cycle duration and thus circumventing the biennial life cycle of sugar beet (Znamenskaya, Zhuzhzhalova, 1989). The totipotency allowed the progeny of the derived lines to preserve genotype, phenotype, ploidy, monogermity, and CMS fixing ability in the field.

To ensure the long-term use of the obtained strain material in breeding practice, the three-year cycle of breeding, biotechnological, and agronomic techniques is used (Kolesnikova, Zhuzhzhalova, 2018). At the first stage, the selected hybrid genotypes are transferred to an in vitro culture and then transplanted to the gene bank of elite clones to ensure the long-term preservation of valuable traits of the material. The second stage includes micropropagation of elite regenerants, rooting, and transplanting into greenhouse soil substrate to form stecklings (small roots) of approximately 100 g in size. At the third stage, the plants are grown for elite seeds. The seeds of a simple hybrid and a heterosis pollinator are harvested separately. This approach has contributed significantly to the development of new-generation high-yielding hybrids as a result of breeding and seed farming efforts at the A.L. Mazlumov All-Russian Research Institute of Sugar Beet and Sugar (Ramon, Voronezh Region) and is applied commercially.

Cell breeding. Breeding at the cellular level includes development of the methods for cultivation of isolated plant cells, organs, and tissues to increase hereditary variability. Through that, the methods involving the introduction of various chemical and physical factors, such as mutagenic substances, genetic constructs with alien genes, selective agents, etc., into culture media stimulate variability in plant organisms and increase their stability against environmental stresses (Dubrovna, 2017; Kourelis, van der Hoorn, 2018).

The experiments showed that exposure to 2 to 6 мМ ethyl methanesulphonate mutagen (EMS) for 30 minutes has a stimulating effect on sugar beet petioles (Hohmann et al., 2005; Vasilchenko et al., 2020b). The diploid material demonstrated high regeneration ability of 38.1–53.1 %, and in haploids it reached 71.3–87.5 %. This method also ensured genetic variability of regenerants by increasing ploidy level of haploids to that of diploids and polyploids. Molecular genetic analysis using the specific SSR marker mSSCIR 47 identified 9 lines, where exposure to mutagen caused genotypic changes. Cultivation of the identified lines under cover made it possible to preserve up to 92 % of microclones and obtain small roots 30 to 89 g in size. (Vasilchenko et al., 2020a). The plant material will be further studied at the A.L. Mazlumov All-Russian Research Institute of Sugar Beet and Sugar using the TILLING method (Targeting Induced Local Lesions in Genomes) developed by C.M. Mc Callum et al. (2000).

The use of genetic constructs Agrobacterium tumefaciens with bacterial genes mf2 (Bacillus thuringensis) and mf3 (Pseudomonas fluorescens) inducing nonspecific resistance against fungal and viral pathogen also proved to be effective (Dzhavakhia et al., 2005; Vasilchenko et al., 2009). Cocultivation of sugar beet tissues and A. tumefaciens with alien genes mf2 and mf3 produced Fusarium rot resistant GM crops, whose transgenic nature was confirmed by molecular and biochemical analysis, as well as by phytopathological assessment under cover (Khussein et al., 2013; Vasilchenko et al., 2014). The total of seven new sugar beet lines with mf2 gene and eight lines with mf3 gene have been derived so far.

The effect of heavy metal ions, particularly Cd2+, on the cultivated seedlings was noteworthy (Cherkasova et al., 2020). Several samplings with cadmium acetate (Cd(CH3CO2)2) content of 4 мМ showed high adaptability and survivability of regenerants up to 74.8 %, which indicated their high resistance. Molecular genetic analysis of these microclones showed the presence of significant SNPs in MTP4, which presumably played a major part in development of resistance against this particular abiotic stress (Nalbandyan et al., 2019; Khussein et al., 2021). The obtained results have allowed researchers at the A.L. Mazlumov All-Russian Research Institute of Sugar Beet and Sugar create and test isogenic lines of sugar beet showing promise in terms of heavy metal resistance.

It is known that abscisic acid (ABA) acts as a stress hormone in plants, causing stomatal conductance and transpiration reduction as a drought resistance mechanism (Lutova et al., 2010). ABA controls abiotic stresses in plants (salinity, drought, and temperature) by putting plant tissues (cells, buds, and leaves) into resting state via stomatal closure. It is also possible that the role of this hormone is to trigger the resistance mechanism, which then develops on its own regardless of the hormone’s content (Titov et al., 2007). This is why the investigation of ABA properties in vitro may facilitate the development of drought-resistant breeding material. The optimal ABA content (2.0–3.0 mg/l) ensuring the ratio of drought-resistant sugar beet regenerants at 70.0–87.5 % has been found in (Cherkasova et al., 2018, 2021). Thus, abscisic acid can be used to simulate droughts, when developing the sugar beet strains tolerant to osmotic stresses.

Improving the adaptive properties of regenerants in the reproductive cycle by increasing their genetic variability makes it possible to obtain new starting material and quickly reproduce it.

## Reproductive ability of generative organs

Self- and cross-incompatibility

Sugar beet is a monoecious hermaphroditic species characterized by cross-pollination and the self-incompatibility preventing self-fertilization. Most plants in sugar beet populations of mixed origin (65.3–78.1 %) are self-incompatible. These plants do not set seeds in isolation after 1–2 inbreedings. The occurrence rate of self-compatible (self-fertile) plants in varietal populations is 4.3–12.9 %, given the pollen grain fertility of up to 83.6–98.2 %. Self-fertile strains are typically characterized by higher crop yields and sugar contents, but some evidence shows that inbreeding depression is not completely suppressed.

Experiments showed that in some strains the selfincompatibility of individual plants was barely affected by environmental changes in cultivation conditions (Kornienko, Znamenskaya, 2001). For instance, plants cultivated in different environmental areas only showed minor differences in seed setting, with the average value of 7.4 % in Ramon (Voronezh Region) and 6.3 % in Przhevalsk (Kyrgyzstan). At the same time, a number of authors demonstrated that seed setting in self-pollinating plants at lower temperatures was significantly higher than that at higher temperatures. This discovery formed the basis for the seed reproduction method at low temperatures used to create a vast collection of inbred sugar beet strains (Maletskii, Maletskaya, 1996). The discrepancies in the results of various studies indicate the complexity of seed reproduction processes in sugar beet. Genetic and physiological mechanisms underlying these processes require additional research and remain relevant in the context of sexual reproduction.

According to the relevant genetic data, the incompatibility in sugar beet is a rather complex gametophytic system based on the complementary interaction of alleles 2–4 of S-loci (Larsen, 1977). These data were taken into consideration in production of strain material by self-pollination with circumvention of physiological self- and cross-incompatibility barriers for successful transition from population breeding to interstrain heterotic hybrid breeding

The primary internal factor affecting the seed setting under inbreeding is genetic and hormonal regulation of the fertilization process. The seed setting in selfcompatible strains remained at the self-pollination level or decreased slightly. Intrastrain crosses in partially compatible strains increased the seed setting by a multiple of 1.5–13. Selection of individual plants from strains of interest differing in pollen recognition by the stigma and pollination ability turned out to be a promising technique as well. This selection has made it possible to produce strain material with prevalent traits of the female or male parent to increase the effectiveness of close (intrastrain) crosses (Kornienko et al., 2014).

To determine the degree of incompatibility and investigate the pollen tube growth, fluorescence microscopy modified for sugar beet studies was used (Zaykovskaya, Zhuzhzhalova, 1976; Vaisman et al., 1984). The method was applied to identify potential seed productivity of plants as early as the flowering stage, and thus made it possible to determine the seed setting and assign pairs while of breeding material hybridization based on pollen tube growth activity. The incompatibility response localization manifested itself clearly in the morphological changes in pollen tubes, thickenings, bulgings, inhibited growth activity at the stigma or in the calcium oxalate deposition area in the ovary. Presumably, Ca++ ions play a major part in pollen tube growth, since it is one of the first pollen germination regulators (Bednarska, 1989). No other element facilitating germination in absence of Ca++ was found.

Based on period of self-incompatibility gene activity and localization of gene products, sporophytic and gametophytic incompatibility types were defined. From the morphological perspective, sporophytic responses are localized at the stigma, and gametophytic ones – in the style. The current concept is that the self-incompatibility response localization in sugar beet matches that in the species with gametophytically controlled self-incompatibility (Vishnyakova, 1998).

Nucleic acid content in ovary and ovule cell nuclei observed by cytophotometry was another indication of incompatibility gene activity in sugar beet (Zhuzhzhalova et al., 2007). Compatible pollination was usually reflected in these cells by increase in nuclear RNA content and total nuclear nucleic acid content. The RNA/DNA ratio under incompatible pollination remained the same as in non-pollinated pistils. A significant increase in RNA content of up to 36 % was recorded after the third day of delayed pollination. It is possible that genetically determined incompatibility mechanism manifesting itself at this stage in DNA molecules indicates functional changes in ovule nuclei associated with fertilization process, as in other plants (Kovaleva, 1991). Thus, we may assume that fertilization stimulates transcription and reduplication processes facilitating increased RNA contents and total nuclear nucleic acid contents. Weakening of the incompatibility barrier correlates with RNA accumulation in cell nuclei. The data obtained also show that genetic and physiological incompatibility properties are currently understudied and still remain a relevant issue.

The further studies showed that pollen tube growth processes were also accompanied by complex hormonal rearrangement in the stigma and style. This is confirmed by the experiments determining contents of endogenous phytohormones, i. e. ethylene, IAA, ABA, gibberellins, and cytokines, in sporophytic tissues of the pistil (Kovaleva et al., 2016). This is why the inner workings of the plant reproduction system, and especially the role of self- and cross-incompatibility, will still be relevant research issues in the near future

The use of this trait was a reasonable way to overcome the incompatibility barrier in order to advance the research of heterosis breeding, including controlled crossses and production of interstrain hybrids using the fertile base

## Cytoplasmic male sterility

Normal (N) and sterile (S) are two types of cytoplasm found in sugar beet, and their effects are regulated by two recessive genes x and z (Оwen, 1952). Complete male sterility is designated as Sxxzz. The presence of dominant genes in the heterozygous state produces semi-sterile forms SXxzz and SXxZz. However, the causes of CMS in sugar beet have not been studied completely and still require more in-depth research (Dymshits et al., 2010).

In the context of breeding, an CMS trait is fixed and maintained in the selected sugar beet strains by inbreeding with an CMS maintainer of high pollen fertility and seed setting (Balkov et al., 2017). Peculiarities of genetic variability in breeding material are identified using DNA markers. Micro-satellite analysis (SSR) made it possible to identify polymorphisms of MS strains, multigerm pollinators (MP) of sugar beet, and their hybrids (Nalbandyan et al., 2021). This analysis was facilitated by determination of polymorphism content (PIC) for each locus. The use of Unigenes SSR markers (9 primer pairs) made it possible to clearly differentiate the material based on calculation of genetic distances and identify parent components best suited for crosses. Primers with the highest SSR polymorphism such as Unigene 22373, Unigene 27833, Unigene 16898, Unigene 7492, Unigene 24552 were recommended for identification of sugar beet breeding materials (Nalbandyan et al., 2021).

The studies into presence of CMS-associated minisatellite locus TRs in the starting material were of interest in terms of sugar beet breeding (Nishizava et al., 2000; Bragin et al., 2011). Experimental screening of the sugar beet breeding material based on mini-satellite loci TR1 and TR3 showed the presence of N- and S- type mitochondrial genomes (Fedulova et al., 2022). MS-plants included amplicons with lengths of 400 bp. О-type pollinator plants (sterility maintainers) were characterized by DNA amplicon lengths of 700 bp. for TR1 primer and 500 bp for TR3. The selected material makes the breeding cycle 2–3 times as short and may therefore become widely used in hybrid breeding.

It was observed in breeding research that male sterility occurring in self-fertile strains after multiple inbreedings manifests itself in formation of pistillate flowers (Oshevnev et al., 1986a, b). Stamens and anthers in these flowers are replaced with pistillate structures, i. e. stigmas or ovules without embryo sacs. Phenotypic ratio determined for the progeny of strain material in process of breeding showed that pistillate trait was controlled monogenically (Oshevnev, Gribanova, 2010). Strains with sterile anthers were used as female parents for interstrain sugar beet hybrids. Fertile plants with high self-compatibility (self-fertility) selected from this material were used as pollen sterility maintainers in hybrid breeding. The scheme for producing O-type strains based on the pistillate trait was the finishing stage of a consistent transition to breeding for heterosis (Oshevnev, Gribanova, 2010).

The results obtained have shown that sterilization of male gametophyte may follow different scenarios, but eventually results in gynodioecy, i. e. the occurrence of plants with hermaphrodite and female flowers in the population. The further investigation of this phenomenon is of great significance for genetic research.

## Apomixis (agamospermy)

The use of apomixis (agamospermy) is among the most significant trends in plant breeding. This reproduction mode with no gamete fusion in some cases ensures maternal inheritance, uniformity of progeny, and high yields in an unlimited line of generations. Thus, the analysis of morphogenetic processes and investigation of embryological development nuances in agamospermous sugar beet strains have a potential to reveal new breeding applications of apomictic reproduction.

Genetic polymorphism of plant reproduction modes with no gamete fusion is typically represented by two main types of apomixis known as gametophytic and sporophytic, and by embryoidogeny, i. e. somatic embryo formation in the ovule or on vegetative organs.

Gametophytic apomixis. Cytological studies showed that sugar beet plants tend to form embryos with haploid set of chromosomes by apogamy from embryo sac nuclei, i. e. synergids or antipodals. However gametophytic apomixis in sugar beet manifested itself in the form of diplospory (Fomenko et al., 2003), which could possibly be explained by the use of breeding material obtained by artificial pollination with pollen exposed to high doses of gamma radiation ranging from 1500 to 3000 Gy (Agafonov et al., 1992). Cytological study of the plants obtained from this material demonstrated that meiosis in megasporogenesis did not produce megaspore tetrads. As a result, embryo sac cells with a nonreduced number of chromosomes formed directly from the megasporocyte, which commonly causes unstable genetic changes. At the same time, the method made it possible to create several sugar beet gamma-strains with signs of facultative apomixis and CMS fixation ability. The hybrids obtained using the apomictic strain γ-МС-2113 showed crop yields of up to 122.2 % and sugar contents of up to 104.5 % compared to the control (Bogomolov, 2010). This plant material is currently used in breeding

Sporophytic apomixis. This apogamy type is based on embryo development from somatic cells of a female parent with a diploid set of chromosomes taken from nucellus, integuments, or endosperm of the female parent or daughter embryo cells created as a result of fertilization (Batygina, 2010). The experiments in sugar beet found multiple instances of budding or ovule splitting into two or more new ones. As a result, several embryos with different inheritance, i. e. a sexual embryo and embryos formed from nucellar or integumentary somatic cells, developed in seeds. The embryos developing from endosperm nuclei were typically observed in the chalazal end of the embryo sac in process of creating polyploid or aneuploid sugar beet strains (Yarmolyuk et al., 1990).

The discovered embryo development peculiarities have shown that coexistence of sexual and asexual modes of seed reproduction leads to significant differences in quality of seed material (Zaykovskaya, Peretyat’ko, 1977). The genetic diversity of strain material in these cases causes some issues for hybrid breeding.

Embrioidogeny. This plant reproduction type represents a special category of vegetative reproduction with embryoid (somatic embryo) as its structural unit. Similarly to sexual embryos, it is an initial stage of a new organism, rather than its part (bud, leaf, or root) as observed in cutting propagation by gemmorhizogenesis (Batygina, 2010). Floral and vegetative embryoidogeny types are defined based on origin and location of somatic embryos on the parent plant.

Floral embryoidogeny in sugar beet represents embryo development from haploid nuclei of the embryo sac (egg cell, synergids, and antipodals) and diploid tissue cells of the ovule (nucellus, integuments, and endosperm). Embryological studies into in vitro cultivation of non-fertilized ovules made it possible to identify the formation stages of haploid embryos (Podvigina, 2010; Tomaszevska-Sowa et al., 2017). It was found that division of haploid cells of the egg apparatus or antipodals produced a multicellular proembryo similar in its development to a sexual embryo. This technique made it possible to obtain genetically and morphologically diverse material with high homozygosity from donor plants (5–6 times as fast). The present studies made it possible to accelerate the creation of homozygous lines, which were then widely used in sugar beet breeding (Batygina, 2010; Kikindonov et al., 2016; Lamaoni et al., 2018; Pazuki et al., 2018).

Vegetative embryoidogeny is based on embryo development from somatic cells of vegetative organs. In vitro cultivation of sugar beat petioles on the Murashige– Skoog medium supplemented with 6-BAP (0.2–0.3 mg/l) and 2.4-D (0.1 mg/l) stimulated morphological development of seedlings from epidermal cells of the petiole similar to formation stages of gametes in embryo sac. At first, formation of one or several initial cells similar to embryo sac gametes was observed. These cells had denser cytoplasm and a large nucleus with a nucleolus. Then, transverse fission of the initial cell occurred forming the future root cells. Subsequent divisions produced embryo-like structures, i. e. embryoids, transforming into seedlings (Bogomolova, Zhuzhzhalova, 1998). The data obtained agree with the findings of foreign authors, who also observed the development of structural elements similar to hypocotyl with leaves from epidermal cells at the adaxial surface of the cultivated sugar beet petioles (Mahmoud et al., 2017).

Parthenogenesis. This apomictic reproduction type implies embryo development from an egg cell not involving a male gamete or from sperm not involving an egg cell (Maletskiy, 2005). According to the relevant concepts, parthenogenetic development of a sugar beet embryo is attributed to epigenetic inheritance and variability. It is associated with the internal or external signals received by ovule cells of flowering plants and making them change their development program. Investigation of sugar beet strains with defected pollen (CMS) in the field showed that their seed reproduction rate was on par with the biparental mode or outperformed it. Parthenogenetic seed progeny demonstrated high occurrence rate of seeds with haploid sets of chromosomes. Haploids amounted to 3–10 % of the total germinated seeds, which is four orders higher than normal and matches the occurrence rate of haploids in the culture medium in vitro (Maletskiy et al., 2015). At the same time, it should be noted that obtaining haploids in parthenogenetic progeny is 3–4 orders cheaper than in vitro.

The further research of parthenogenesis may show new opportunities in commercial breeding due to the significant simplification and cost-effectiveness of MShybrid breeding processes. Thus, we may assume that this way of obtaining haploids may become one of the most efficient sugar beet breeding techniques. Initiation of various reproduction modes in sugar beet plants is ensured by potential capabilities of the reproductive system of the species, which makes it possible to maintain species and population homeostasis in general.

## Conclusion

The wide variety of ways to develop new traits and attributes in plants is the key feature of reproductive biology techniques for sugar beet. The pregenerative development stage turned out to be best suited for vegetative development and production of a new breeding material capable of maintaining valuable traits using particulation and micropropagation. These methods are backup reproduction mechanisms, which, combined with seed reproduction, ensure the organism’s flexibility and make it possible to preserve the genotype, phenotype, ploidy level, and other valuable traits in the field

Cell breeding methods produce the material with altered ploidy that is resistant against environmental stresses. Morphogenetic reproduction techniques producing strain material with modified traits and facilitating the transition from population to hybrid breeding may be considered the best studied ones. A number of breeding processes and mechanisms, such as self- and cross-incompatibility, CMS, and apomixis have contributed significantly to the development of various trends in reproduction systems. Experimental studies based around apogamy, diplospory, and parthenogenesis have demonstrated new opportunities in commercial breeding and biotechnology. However, the agamospermy methods remain insufficiently studied to be applied in commercial breeding.

The studies into apomictic reproduction of sugar beet have shown the most promise in plants with CMS due to the fact that inheritance of individual traits in hybrid progeny is driven by gene transfer from female parents. Cytoplasmic male sterility is accompanied by the presence of two types of intracellular organelles in cytoplasm (mitochondria and chloroplasts with their own genetic material in the for of mtDNA and cpDNA). Cytological and molecular genetic studies of sugar beet with CMS are of interest, because genotypic and phenotypic polymorphism can be identified in agamospermous progeny. This will open new opportunities for applying innovative technologies to produce homozygous sugar beet material with new traits for breeding purposes.

## Conflict of interest

The authors declare no conflict of interest.
